# Increased shedding of HU177 correlates with worse prognosis in primary melanoma

**DOI:** 10.1186/1479-5876-8-19

**Published:** 2010-02-23

**Authors:** Heather K Hamilton, Amy E Rose, Paul J Christos, Richard L Shapiro, Russell S Berman, Madhu Mazumdar, Michelle W Ma, Daniel Krich, Leonard Liebes, Peter C Brooks, Iman Osman

**Affiliations:** 1 Department of Dermatology, New York University School of Medicine, New York, NY, USA; 2 Division of Biostatistics and Epidemiology, Weill Medical College of Cornell University, New York, NY, USA; 3 Department of Surgery, New York University School of Medicine, New York, NY, USA; 4 Department of Medicine, New York University School of Medicine, New York, NY, USA; 5 Departments of Radiation Oncology and Cell Biology, New York University School of Medicine, New York, NY, USA; 6 Maine Medical Center Research Institute, Center for Molecular Medicine, 81 Research Drive Scarborough, ME 04074, USA

## Abstract

**Background:**

Increased levels of cryptic collagen epitope HU177 in the sera of melanoma patients have been shown to be associated with thicker primary melanomas and with the nodular histologic subtype. In this study, we investigate the association between HU177 shedding in the sera and clinical outcome in terms of disease-free survival (DFS) and overall survival (OS).

**Methods:**

Serum samples from 209 patients with primary melanoma prospectively enrolled in the Interdisciplinary Melanoma Cooperative Group at the New York University Langone Medical Center (mean age = 58, mean thickness = 2.09 mm, stage I = 136, stage II = 41, stage III = 32, median follow-up = 54.9 months) were analyzed for HU177 concentration using a validated ELISA assay. HU177 serum levels at the time of diagnosis were used to divide the study cohort into two groups: low and high HU177. DFS and OS were estimated by Kaplan-Meier survival analysis, and the log-rank test was used to compare DFS and OS between the two HU177 groups. Multivariate Cox proportional hazards regression models were employed to examine the independent effect of HU177 category on DFS and OS.

**Results:**

HU177 sera concentrations ranged from 0-139.8 ng/ml (mean and median of 6.2 ng/ml and 3.7 ng/ml, respectively). Thirty-eight of the 209 (18%) patients developed recurrences, and 34 of the 209 (16%) patients died during follow-up. Higher HU177 serum level was associated with an increased rate of melanoma recurrence (p = 0.04) and with increasing mortality (p = 0.01). The association with overall survival remained statistically significant after controlling for thickness and histologic subtype in a multivariate model (p = 0.035).

**Conclusions:**

Increased shedding of HU177 in the serum of primary melanoma patients is associated with poor prognosis. Further studies are warranted to determine the clinical utility of HU177 in risk stratification compared to the current standard of care.

## Background

Limitations of the current melanoma staging paradigm beget limitations in our ability to determine the most appropriate treatment for primary melanoma patients with regard to maximizing therapeutic benefit and minimizing morbidity. Well-characterized clinical prognostic markers such as tumor thickness and ulceration only partly explain the variability in the clinical course of melanoma. Patients with thin melanoma <1 mm, characterized as having a favorable prognosis, have reported rates of metastasis ranging from 3-22% [1]. Conversely, patients with thicker lesions not uncommonly have extended periods of disease-free survival. Although sentinel lymph node biopsy has improved our ability to predict prognosis for patients with intermediate thickness lesions, further markers are needed to determine which of these patients are most likely to develop metastases and thus are most likely to benefit from post-surgical adjuvant therapy.

There is a need for development of new biomarkers that reflect the underlying melanoma biology. Mitotic rate has recently become part of the American Joint Committee on Cancer staging criteria based on studies demonstrating that its addition to a morphologically-based classification system improved risk stratification for patients with thin primary melanoma [2]. Advances in the understanding of melanoma biology have resulted in the discovery of other promising protein biomarkers that are predictive of melanoma-specific mortality and reflective of varying aspects of tumorigenesis including resistance to antigrowth signals (p16/INK4a), limitless replicative potential (Ki-67), tissue invasion (matrix metalloproteinase-2), and sustained angiogenesis (iNOS) [3]. None of these biomarkers, however, have been adopted into clinical practice which may be attributable to several reasons including lack of multivariate analyses with subsequent overestimation of prognostic utility [3].

Recent efforts in genomics research have focused on the development of tumor specific and patient specific gene expression signatures that are predictive of clinical outcome or response to treatment. Even in large scale studies, however, the prognostic accuracy of gene classifiers has not yet proven to be superior to thickness and ulceration in predicting metastasis [4]. Furthermore, gene expression profiling typically requires fresh frozen tissue from the surgical resection, and studies of the effect of sampling melanocytic lesions for research have raised concerns about the possibility of compromising the accuracy of the pathologic diagnosis and subsequent staging [5]. At present, the emerging technology is labor-intensive and likely prohibitively expensive for integration into the common clinical practice for melanoma patients. Immunohistochemistry-based biomarkers are also limited by experimental variability, lack of reproducibility, and inter-observer variation in the classification of staining intensities [6]. By contrast, serum-based biomarkers are non-invasive, relatively low cost, and can easily be incorporated into clinical practice as a way to monitor disease progression over time.

It is known that cellular interactions with the extracellular matrix (ECM) can regulate a wide range of biologic functions including adhesion, migration, proliferation, and angiogenesis [7]. Previous studies have identified cryptic regulatory epitopes that, under normal physiologic conditions, are hidden within the 3-dimensional structure of the ECM protein collagen [8,9]. Following proteolytic remodeling of the collagenous ECM during tumor growth and invasion, however, these unique cryptic epitopes are exposed and shed into the serum. Cryptic collagen epitope HU177 has been specifically associated with increased angiogenesis and tumor growth *in vivo *[9]. We have successfully developed an ELISA assay to detect and quantify levels of cryptic epitope HU177 in the serum of melanoma patients and demonstrated that the level of HU177 correlated with tumor thickness and with the nodular histologic subtype [10]. In the current study, we sought to determine the prognostic relevance of HU177 serum levels. We demonstrate that HU177 shedding in the sera is associated with increased recurrence and decreased overall survival independent of tumor thickness suggesting that it may have potential as a biomarker of aggressive disease in primary melanoma. Additionally, HU177 serum levels may be useful in the stratification of patients for inclusion in clinical trials of anti-angiogenesis based chemotherapeutics.

## Methods

The study cohort consisted of 209 primary melanoma patients prospectively enrolled in the Interdisciplinary Melanoma Cooperative Group (IMCG) at the New York University (NYU) Langone Medical Center between September 2002 and November 2006. Demographic and clinicopathologic data were recorded prospectively for all patients, and patients were followed through July 2008. Follow-up ended in July 2008 to allow sufficient time for data auditing, which was completed by December 2008. The NYU Institutional Review Board approved this study and informed consent was obtained from all patients at the time of enrollment.

All blood samples were collected at the time of primary melanoma diagnosis in 10 ml BD serum tubes, stored immediately at 4°C, and then centrifuged at 10°C for 10 minutes at 1,500 × g. In 178 patients, serum was collected after surgery. In 29 patients, serum was collected on the day of surgery, and in 2 patients, serum was collected before surgery. Previously published results demonstrated that time of collection does not influence the relationship between HU177 level and tumor characteristics [10]. The supernatant serum was aliquoted into 1.5 ml cryovials and stored at -80°C until further use. All samples studied with the ELISA assay were subjected to only one freeze-thaw cycle.

HU177 cryptic epitope concentration (ng/ml) was quantified by a capture assay described in detail previously [10]. Briefly, 96-well microtiter plates were coated with a monoclonal antibody to HU177. Patient samples and denatured collagen IV standards were incubated in each well in triplicate, followed by incubation with biotinylated anti-collagen IV antibody (Southern Biotech, Birmingham, Alabama), subsequently with anti-biotin monoclonal antibody conjugated to horseradish peroxidase (Sigma Aldrich, St. Louis, Missouri), and lastly with 3, 3',5,5'-tetramethylbenzidine (TMB) substrate. Substrate absorbance was measured at 400 nm using a model 680 Bio-Rad microplate reader (Bio-Rad Laboratories, Hercules, California). Although there is no true positive or negative with which to determine the sensitivity and the specificity of the assay, the accuracy of the levels was determined using a standard curve of known concentrations of denatured collagen that ranged from 0-40 ng/ml and fit with either a linear or a second degree polynomial equation (r^2 ^≥ 0.993) from which the concentration of cryptic epitope in patient samples was extrapolated [10]. Random samples were also subjected to additions of 100 ng denatured collagen and recoveries were equal to the endogenous level plus the external spike. Investigators performing the HU177 ELISA assay were blinded to clinicopathologic data.

Descriptive statistics were calculated for baseline demographic and clinicopathologic characteristics. HU177 values were dichotomized into two groups using the mean (6.2 ng/ml) and median (3.7 ng/ml) values determined previously in this cohort [10]. The chi-square test or Fisher's exact test, as appropriate, was used to compare recurrence and mortality proportions between the two HU177 categories. Disease-free survival (DFS) and overall survival (OS) were estimated by Kaplan-Meier survival analysis and the log-rank test was used to compare DFS and OS between the two HU177 groups. Multivariate Cox proportional hazards regression models were employed to examine the effect of HU177 category (e.g. ≤ 3.7 ng/ml vs. >3.7 ng/ml) on DFS and OS, adjusting for tumor thickness (continuous), histologic subtype (nodular/other melanoma vs. superficial spreading melanoma), and ulceration status. The proportional hazards assumption was evaluated by statistically assessing the interaction of each predictor variable with time in the model. In addition, Schoenfeld residuals for each predictor variable in the model were examined when evaluating the proportional hazards assumption. All p-values were two-sided with statistical significance evaluated at the 0.05 alpha level. Ninety-five percent confidence intervals (95% CI) were calculated to assess the precision of the obtained estimates. All analyses were performed in SAS Version 9.1 (SAS Institute Inc., Cary, North Carolina) and Stata Version 10.0 (Stata Corporation, College Station, Texas).

## Results

Clinical and pathologic characteristics of the 209 patients in the study population are presented in Table 1. The median follow-up time for survivors was 54.9 months. Follow-up ranged from 2 months to 81 months, with the lower end resulting from loss to follow-up or study withdrawal prior to the end of the study period. Thirty-eight of the 209 (18%) patients developed recurrences and/or metastases (13 skin, 8 lymph node, 17 visceral), and 34 of the 209 (16%) patients died during follow-up. The mean and median HU177 levels (ng/ml) for the entire cohort were 6.2 and 3.7 (range 0.003-139.8), respectively. The number of recurrences, deaths, and median HU177 levels by melanoma stage are displayed in Table 2.

**Table 1:** Baseline characteristics of 209 primary melanoma patients

Variable	Patients (n = 209)
	Number (%)
Gender	
Male	124 (59.3)
Female	85 (40.7)
	
Age (years)	
Mean ± SD; Median	58.3 ± 16.9; 58
	
Primary tumor histologic subtype	
Superficial spreading melanoma	123 (58.9)
Nodular melanoma	52 (24.9)
Acral lentiginous melanoma	6 (2.9)
Desmoplastic melanoma	6 (2.9)
Lentigo maligna melanoma	7 (3.3)
Other melanoma	10 (4.8)
Unknown	5 (2.4)
	
Thickness (mm)	
Mean ± SD; Median	2.09 ± 3.83; 0.95
	
Ulceration	
Absent	169 (80.9)
Present	35 (16.7)
Unknown	5 (2.4)
	
AJCC stage	
Stage I	136 (65.0)
Stage II	41 (20.0)
Stage III	32 (15.0)

Abbreviations used: SD, standard deviation; AJCC, American Joint Committee on Cancer.

**Table 2:** Recurrences, deaths, and median HU177 levels by stage

Stage	Recurrences	Deaths	Median HU177 (ng/ml)
I (n = 136)	9 (7%)	9 (7%)	3.66
II (n = 41)	11 (27%)	10 (24%)	3.91
III (n = 32)	18 (56%)	15 (47%)	3.89

**Figure 1:**
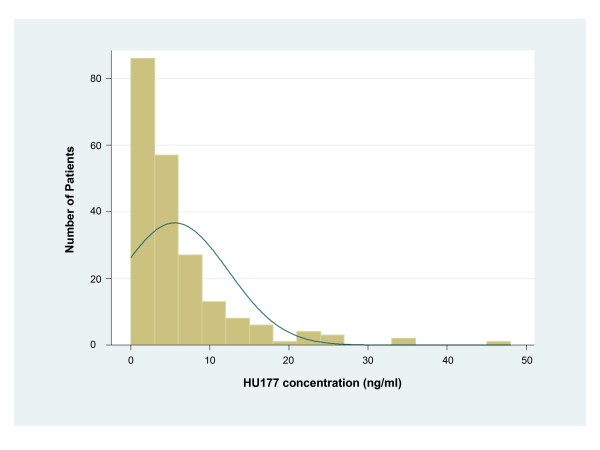
**Histogram of HU177 sera concentration in 209 patients with primary melanoma**. Median = 3.7 ng/ml, Mean = 6.2 ng/ml, SD = 11.5 ng/ml, Min = 0.003 ng/ml, Max = 139.8 ng/ml. One patient with a HU177 concentration of 139.8 ng/ml is not shown.

The HU177 level was greater than the mean HU177 level of the cohort (6.2 ng/ml) in 59 patients (28%) and greater than the median concentration (3.7 ng/ml) in 106 patients (51%) (Figure 1). Because the distribution of HU177 levels was positively skewed, we analyzed the data using the median in addition to the mean. Analyses based on both mean and median HU177 concentration are provided to allow for a comparison of the two distinct cut points. However, the use of the median HU177 value as a categorical cut point is emphasized in our results.

### Elevated HU177 concentration is associated with increased melanoma recurrence

**Figure 2:**
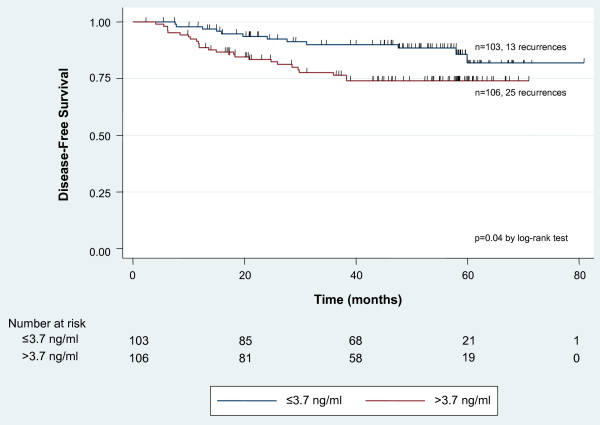
**Kaplan-Meier analysis for disease-free survival by median epitope concentration**. Patients with elevated HU177 concentrations above the median value demonstrated a reduced disease-free survival probability compared to patients with HU177 concentrations below the median (HU177 >3.7 ng/ml: n = 106 patients, 25 recurrences; HU177 = 3.7 ng/ml: n = 103 patients, 13 recurrences; p = 0.04 by log-rank test).

HU177 sera concentration greater than the median (3.7 ng/ml) was associated with a higher recurrence rate compared to HU177 sera concentration less than or equal to the median (23.6% vs. 12.6%; p = 0.04). This association remained statistically significant when the mean (6.2 ng/ml) was used to dichotomize the HU177 distribution (27.1% vs. 14.7%; p = 0.04). Kaplan-Meier survival analysis demonstrated improved DFS for patients with HU177 sera concentration less than or equal to the median compared to patients with sera concentration greater than the median (p = 0.04 by log-rank test) (Figure 2).

### Elevated HU177 concentration is associated with increasing mortality

**Figure 3:**
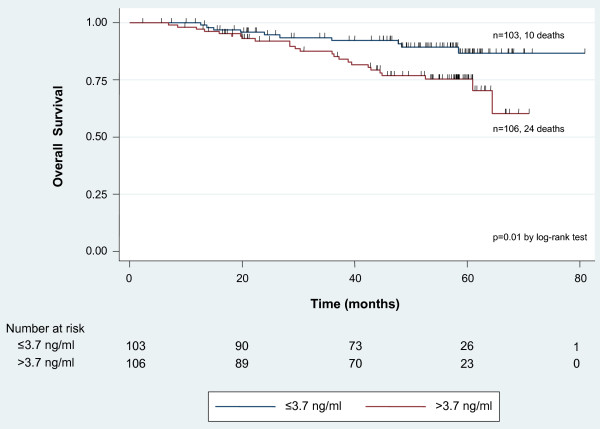
**Kaplan-Meier analysis for overall survival by median epitope concentration**. Patients with elevated HU177 concentrations above the median value demonstrated a reduced overall survival probability compared to patients with HU177 concentrations below the median (HU177 >3.7 ng/ml: n = 106 patients, 24 deaths; HU177 = 3.7 ng/ml: n = 103 patients, 10 deaths; p = 0.01 by log-rank test).

HU177 sera concentration greater than the median (3.7 ng/ml) was associated with a higher mortality rate compared to HU177 sera concentration less than or equal to the median (22.6% vs. 9.7%; p = 0.01). The observed association remained statistically significant when the mean HU177 level was used to dichotomize the HU177 distribution (28.8% vs. 11.3%; p = 0.002). Kaplan-Meier survival analysis demonstrated improved OS for patients with HU177 sera concentration less than or equal to the median compared to patients with sera concentration greater than the median (p = 0.01 by log-rank test) (Figure 3).

### HU177 concentration is associated with disease-free and overall survival after adjustment for tumor thickness and histologic subtype

Because the number of recurrences in the cohort was relatively low (n = 38), the most balanced multivariate model included 3 variables inclusive of the epitope concentration. Variables that were most strongly correlated with epitope concentration in the univariate analyses (histologic subtype and thickness) were included in the multivariate model. High levels of HU177 remained an independent prognostic factor for DFS and OS when controlling for tumor thickness and for histologic subtype. In the DFS hazard model controlling for tumor thickness and histology, the hazard ratio for HU177 >3.7 ng/ml (the median) was 2.01 (95% CI = 1.002, 4.04; p = 0.049) (Table 3). In the OS hazard model controlling for tumor thickness and histology, the hazard ratio for HU177 >3.7 ng/ml (the median) was 2.23 (95% CI = 1.06, 4.70; p = 0.035) (Table 3). The proportional hazards assumption was not violated for any of the predictor variables in the DFS and OS models.

**Table 3:** Association between HU177 concentration and DFS/OS, controlling for tumor thickness and histologic subtype

Variable	P-value	Hazard ratio (95% CI)
Disease-free survival		
Epitope concentration^a^	0.049	2.01 (1.002, 4.04)
Tumor thickness^b^	0.065	1.05 (1.00, 1.10)
Histology^c^	0.001	3.66 (1.70, 7.86)
		
Overall survival		
Epitope concentration^a^	0.035	2.23 (1.06, 4.70)
Tumor thickness^b^	0.004	1.08 (1.02, 1.13)
Histology^c^	0.363	1.41 (0.67, 2.98)

Abbreviations used: DFS, disease-free survival; OS, overall survival; CI, confidence interval.

^a ^>3.7 ng/ml vs. ≤ 3.7 ng/ml (referent).

^b ^Treated as a continuous variable in the model.

^c ^Nodular/other melanoma vs. superficial spreading melanoma (referent).

If ulceration is included in the multivariate model (instead of histologic subtype), the independent prognostic value of HU177 level remains statistically significant (DFS, p = 0.048; OS, p = 0.048), and tumor thickness loses its predictive significance (DFS, p = 0.257; OS, p = 0.199) (not shown). This suggests that the variables are collinear and thus only one should be added to the model. Because thickness was more closely associated with epitope concentration than ulceration in the univariate analysis, it was entered into the multivariate model along with histologic subtype.

Regarding the impact of sentinel lymph node (SLN) data, only 100/209 (48%) patients had SLN biopsies performed, thus its influence on survival could only be meaningfully assessed on a univariate analysis. A subset analysis, however, of the 100 patients who underwent SLN biopsy showed that SLN status was a significant predictor of both DFS (HR 3.73, 95% CI = 1.75-7.94; p = 0.0006) and OS (HR 2.58, 95% CI = 1.00-6.68; p = 0.05) on univariate analysis.

## Discussion

Our results suggest that pro-angiogenic cryptic collagen epitope HU177 may have prognostic significance as a biomarker of poor outcome in primary melanoma. Higher levels of HU177 were associated with an increased rate of recurrence and increasing mortality.

Clinical decision making in the care of melanoma patients is based primarily on tumor morphology as thickness and ulceration consistently prove to be the most accurate predictors of survival [11]. Sentinel lymph node biopsy has been shown to be predictive of recurrence, but it is typically only considered standard of care for patients with intermediate thickness lesions. Our previously reported meta-analysis demonstrated that few patients with thin melanoma have a positive SLN, and there are no clinical or histopathologic criteria that can reliably identify thin melanoma patients who might benefit from this intervention [12]. As reflected in our cohort in which 51% of patients have melanomas <1 mm thick, trends in downward stage migration mean that a larger percentage of newly diagnosed melanoma patients will not be considered for SLN biopsy but could nonetheless benefit from non-invasive serologic prognostic markers.

A number of sera markers have been evaluated for their prognostic significance in primary melanoma with limited success. For example, angiogenic factors vascular endothelial growth factor (VEGF), basic fibroblast growth factor (bFGF), interleukin-8 (IL-8), and angiogenin have been studied for their value in predicting outcome. One study reported that elevated concentrations of VEGF independently correlated with poor overall survival [13]. The results, however, have not been replicated by other investigators [14,15]. Similarly, IL-8 and bFGF were found to be independent predictors of overall survival [13], but additional studies to validate their findings are pending. Angiogenin showed less promise: serum levels were not found to correlate with outcome [13]. Other candidates such as S-100 beta, a well-established diagnostic marker for melanoma by immunohistochemistry, have been found to have limited prognostic relevance in early stage melanoma [16].

A serum-based marker of aggressive biology such as HU177 has the potential to identify primary melanoma patients at high risk for the development of distant metastases who should be treated in the post-surgical adjuvant period. Even if the appropriate risk stratification tools were developed, however, current data suggest that adjuvant therapy with interferon fails to confer a survival advantage [17]. Thus, it is imperative that the development of prognostic biomarkers and the development of novel molecularly targeted therapy occur simultaneously. Our results showing a correlation between pro-angiogenic collagen epitope HU177 and worse overall survival suggest that targeting angiogenesis in the post-surgical adjuvant period may be a rational approach for patients with primary melanoma. A shift in the balance between pro- and anti-angiogenic peptides towards angiogenesis promotes neovascularization, which is essential for tumor progression among other processes. Angiogenesis has been successfully targeted in other malignancies, resulting in the FDA approval of anti-VEGF agent bevacizumab for use as combination therapy in the treatment of metastatic colorectal and non-small cell lung cancer [18,19]. The utility of anti-angiogenic therapy in melanoma, however, has not been clearly defined. Since metastatic melanoma has a poor prognosis, anti-angiogenic treatments would delay melanoma progression and have a great impact on cancer-specific mortality. We have already shown the potential utility of HU177 in prognosis but it may also serve as a therapeutic target, similar to bevacizumab but with its effect prior to metastasis. Metastasis requires changes in the vascular basement membrane, of which type IV collagen is a part. Both the pro-angiogenic factor HU177 and the angiogenesis inhibitor tumstatin are type IV collagen cleavage products. Disruption of this balance between pro- and anti-angiogenic peptides promotes neovascularization. Treatments targeting pro-angiogenic factors, such as HU177, appear to be more clinically relevant. A recent study demonstrated that tumstatin slows tumor growth in renal cell carcinoma and colorectal cancer cell lines, but all tumors eventually escaped tumstatin-induced growth inhibition and entered into an exponential growth phase. This rapid growth was shown to result from an up-regulation of genes encoding pro-angiogenic peptides, possibly in response to hypoxic conditions. Genes encoding anti-angiogenic factors were not silenced [20]. Another study investigating carboplatin/paclitaxel/bevacizumab combination therapy in stage IV melanoma demonstrated that the addition of bevacizumab was well tolerated and the median overall survival was higher than in previous reports of single agent treatment with dacarbazine (52 weeks vs. 25.6 weeks) [21]. Although limited conclusions can be drawn from this uncontrolled trial, the results do suggest that targeting angiogenesis, in particular pro-angiogenic factors, as part of a combination chemotherapy regimen may be a useful strategy.

The association between pro-angiogenic HU177 and poor prognosis in our study is consistent with other serum biomarker studies that have identified VEGF and serum angiopoietin-2 (sAng-2) as useful predictors of response to therapy. In a study of 59 patients with metastatic melanoma or renal cell carcinoma receiving high dose recombinant interleukin-2 (IL-2), serum was collected and analyzed for potential biomarkers of response using a customized protein array platform. Serum VEGF and fibronectin were shown to be independently predictive of response to IL-2 [22]. Another serum biomarker study of 98 patients with stage I-IV melanoma identified an increase in sAng-2 levels by 50-400% in 90% of patients during progression from stage III to IV melanoma leading authors to conclude that sAng-2 levels are associated with disease progression in metastatic melanoma [23]. Both of these studies, however, are focused on biomarkers of advanced disease. A notable advantage of our study is that 65% of patients included had stage I melanoma, and the level of HU177 shedding in the serum was predictive of decreased overall survival independent of tumor thickness. Because HU177 has potential as a biomarker that can be utilized early in the disease course, there is perhaps a greater chance that it will influence the clinical decisions that alter the disease course and ultimately impact outcomes.

Our findings emphasize the role of interactions with the cellular microenvironment as potential targets for therapy and biomarker development. A key limitation of current *in vitro *and *in vivo *models is that they often overlook the contribution of the ECM and the tumor microenvironment toward the initiation and progression of tumorigenesis. Increasing evidence, however, supports the notion that melanoma cells interact with the adjacent microenvironment in a bi-directional manner through molecular signals that can modulate the malignant phenotype [24]. Previous *in vivo *studies of HU177 demonstrated that cleavage of type IV collagen during ECM remodeling led to exposure of cryptic regulatory sites, such as HU177, and that an antibody directed at the HU177 cryptic site inhibited cell adhesion, migration, and proliferation on denatured collagen type IV [25]. It is thought that the HU177 measured in sera is shed not from the tumor but from the tumor microenvironment. Thus, while current efforts to target VEGF and other pro-angiogenic factors whose expression is regulated by the melanoma cell have thus far been unsuccessful, our approach focused on non-cellular epitopes as new targets for biomarkers and treatment is novel and highly selective. Preliminary data from pre-clinical trials demonstrate that anti-HU177 mAB TRC093 significantly enhances the anti-tumor activity of bevacizumab in a melanoma mouse xenograft model demonstrating the potential utility of monitoring HU177 as part of an anti-angiogenic therapeutic strategy [26].

We demonstrate that HU177 levels are associated with worse outcome independent of tumor thickness. These results emphasize that, while the shedding of HU177 is associated with tumor remodeling and invasion, it is not merely a surrogate read-out of thickness. In the multivariate model, although the p-value for tumor thickness is lower than that for epitope concentration, the hazard ratio for the epitope concentration is more than double that of thickness (Table 3). Because thickness was analyzed as a continuous variable and HU177 epitope concentration was evaluated as a categorical variable (high vs. low), a true comparison between the strength of these two prognostic factors cannot be undertaken. The analysis demonstrates, however, that HU177 maintains its prognostic value independent of well-characterized prognostic variables that constitute the current standard of care.

## Conclusions

High levels of cryptic collagen epitope HU177 are associated with higher recurrence rates and increasing mortality. HU177 shows promise as a serum biomarker that is reflective of melanoma biology, that can be easily integrated into the clinical management of melanoma patients, and which may have potential as a molecular target for adjuvant therapy. These data justify further validation studies in a larger, independent cohort.

## Competing interests

PCB serves as a consultant to TRACON Pharma and has received honoraria from TRACON Pharma in the last two years.

## Authors' contributions

HKH participated in study design, coordination, data collection, data analysis, and drafting of the manuscript. AER participated in data collection, analysis, drafting, and finalizing the manuscript text. PJC performed the statistical design and analysis under the guidance of MM. RLS recruited patients for the study, provided input on study design, and helped to draft the manuscript. RSB recruited patients for the study, provided input on study design, and helped to draft the manuscript. MM was the principal statistician for the study, providing guidance on statistical design and data analysis. MWM participated in data analysis and manuscript drafting/finalization. DK collected and helped analyze clinical data extracted from the melanoma database. LL participated in the conceptual study design and provided guidance regarding interpretation of results. PCB provided guidance in study design and interpretation of results. IO served as the principal investigator for the project, overseeing the study design, analysis of data, interpretation of results, and writing of the manuscript. All authors read and approved the final manuscript.
